# Therapeutic Effect of Polysaccharide of Large Yellow Croaker Swim Bladder on Lupus Nephritis of Mice

**DOI:** 10.3390/nu6031223

**Published:** 2014-03-24

**Authors:** Xianhong Jiang, Xin Zhao, Huali Luo, Kai Zhu

**Affiliations:** 1 Department of Nephrology, Yongchuan Hospital Chongqing Medical University, Chongqing 402160, China; E-Mails: cqjiangxh@gmail.com (X.J.); cqluohl@gmail.com (H.L.); 2 Department of Biological and Chemical Engineering, Chongqing University of Education, Chongqing 400067, China; E-Mail: foods@china.com.cn

**Keywords:** polysaccharide, large yellow croaker, lupus nephritis, cytokine, mice

## Abstract

The therapeutic effect of polysaccharide of large yellow croaker swim bladder (PLYCSB) on lupus nephritis has been studied *in vivo*. A high concentration (50 mg/kg dose) of PLYCSB reduced the levels of serum inflammatory cytokine levels of IL-6, IL-12, TNF-α and IFN-γ compared to a low concentration (25 mg/kg dose) and control mice. SCr, BUN, TC and TG serum levels of PLYCSB treated mice were lower than those of control mice, and TP and ALB serum levels were higher than control mice. Control mice tested ds-DNA positive at the 6th week, and 50 mg/kg treated mice tested at the 10th week after the experiment began. The output of urine protein of 50 mg/kg PLYCSB treated mice was most closely comparable to the normal mice. The glomerular number of 50 mg/kg PLYCSB treated mice was more than the 25 mg/kg dose and control groups, and the 50 mg/kg dose group showed the lowest glomerular sclerosis index in lupus nephritis mice. By RT-PCR and western blot assay, PLYCSB significantly induced inflammation in kidney tissues of mice by downregulating NF-κB-p65, TGF-β1, Fas, FasL and upregulating IκB-α. These results suggest that PLYCSB showed a potential curative effect on lupus nephritis as a drug or functional food.

## 1. Introduction

As one of the main commercial fishes in the coastal waters of China, the large yellow croaker (*Larimichthys crocea*) swim bladder (LYCSB) contains many nutrients, such as numerous proteins, microelements and vitamins. Traditional medicine advocates that it has a good curative effect on diseases including amnesia, insomnia, dizzy, anepithymia and weakness after delivery [1]. Researchers also suggested that LYCSB served to remove free radicals and ward against inflammation and cancer [2]. LYCSB is an important balancing organ and the amount of polysaccharide in it is as much as 10% of its weight. Polysaccharide is a kind of important functional material. It hass been proven that polysaccharide in swimming bladders can rapidly heal cuts and prevent infection as well as thrombotic events [3]. *In vivo* experiments have proved that polysaccharide in lentinus edodes and spirulina seaweed serves to prevent and cure injuries [4,5].

Lupus nephritis (LN) is systemic lupus erythematosus (SLE). It is a kind of immune complex nephritis caused by exhausting the kidney. Its clinical features include all features of glomerulonephritis [6]. The murine lupus nephritis model is an internationally recognized and widely applied animal model. The experimental conditions are easy to control. Renal pathological lesions appear after the induction. The lesion is similar to SLE occuring in human beings. The main features include lymphonodular hyperplasia, the formation of autoantibodies, and infection with glomerulonephritis [7]. Renal morphologic changes include the segmental or pervasive proliferation of glomerular mesangial, membraneous nephritis and glomerular sclerosis, which is likely to happen in patients with more serious cases [8].

In the present study, the preventative effect on lupus nephritis of polysaccharide of large yellow croaker swim bladder (PLYCSB) was determined. The serum levels and inflammation-related cytokines levels were used to determine the therapeutic effect of PLYCSB on lupus nephritis mice. The status of glomerular tissues was checked by histology. The mRNA and protein gene expressions by the tissues were also assessed to try to understand the basis for the therapeutic effect.

## 2. Materials and Methods

### 2.1. Polysaccharide of Large Yellow Croaker Swim Bladder (PLYCSB) Preparation

Wild Yellow Sea large yellow croaker was purchased from Shandong Province in China. Swim bladder of large yellow croaker (1 kg) was dried by freeze-drying, and the dried samples were crushed. 3 L petroleum ether was added into swimming bladder of large yellow croaker and then reflux extraction was done twice (1 h each time) at 60 °C to remove the protein, and the residuals were gathered after filtration. After this, 3 L ethyl alcohol was added and reflux extraction were performed for 3 h, the residuals without protein were filtered and gathered. Finally, 3 L water was added and the residuals were extracted at 60 °C for 2 h, and the filter liquor was collected. The crude polysaccharide of large yellow croaker swim bladder was collected after evaporating [9].

### 2.2. Lupus Nephritis Experiment

Eight-week-old male DBA/2 mice (*n* = 20) and female B6D2F1 (C57BL/6J × DBA/2) hybrid mice (*n* = 40) were purchased from the Experimental Animal Center of Third Military Medical University (Chongqing, China). They were maintained in a temperature-controlled facility (temperature 23 ± 1 °C, relative humidity 50 ± 5%) with a 12-h light/dark cycle. The mice had unlimited access to a standard mouse chow diet and water. The spleen, thymus and lymphonodus of DBA/2 mice were isolated in a germ-free state. Then, they were grinded and cut into pieces in normal saline. Cells were isolated using lymphocyte isolation medium. A single-cell suspension was made with *D*-Hanks solution. The ratio of spleen, thymus and lymphonodus was 3:2:1. The cell suspension was blended with 2.5 × 10^8^ cells/mL of living cells on day 0, the 3rd day, the 7th day, the 10th day respectively to B6D2F1 mice in the control group and the PLYCSB treated groups. 0.2 mL of cell suspension was injected into the body of B6D2F1 (C57BL/6J × DBA/2) hybrid mice by means of tail vein injection, and then *D*-Hanks solution was injected into mice in the normal group. The control and normal groups B6D2F1 mice received no PLYCSB treatment. After the single-cell suspension was injected at the first day, the PLYCSB groups B6D2F1 mice were received by oral administration of 25 or 50 mg/kg PLYCSB everyday for 12 weeks. These experiments followed a protocol approved by the Animal Ethics Committee of Chongqing Medical University (Chongqing, China) [10].

### 2.3. Analysis of Inflammation-Related Cytokines in Serum by Enzyme-Linked Immunosorbent Assay (ELISA)

For the serum cytokine assay, blood from the inferior vena cava was collected in a tube and centrifuged at 730× *g*, 4 °C for 10 min. The serum was aspirated and assayed as described below. Concentrations of inflammatory-related cytokines IL-6, IL-12, TNF-α and IFN-γ in serum were measured by ELISA according to the manufacturer’s instructions (Biolegend, San Diego, CA, USA). Briefly, biotinylated antibody reagent was added to 96-well plates, then supernatants of homogenized serum were added and the plates were incubated at 37 °C in CO_2_ for 2 h. After washing with PBS, streptavidin-horseradish peroxidase (HRP) solution was added and the plate was incubated for 30 min at room temperature. The absorbance was measured at 450 nm using a microplate reader (iMark; Bio-Rad, Hercules, CA, USA) [11].

### 2.4. Serum Levels of SCr (Serum Creatinine), BUN (Blood Urea Nitrogen), TC (Total Cholesterol), TG (Triglyceride), TP (Total protein) and ALB (Albumin) Determination

Blood was drawn from eye orbit with glass capillary syringe to prepare blood serum every two weeks since the injection of lymphocyte for the first time. The mice blood was collected in a tube and centrifuged at 730× *g*, 4 °C for 10 min. Then the SCr, BUN, TC (total cholesterol), TG, TP and ALB levels of the serum were determined using commercially available kits (Unison Biotech Inc., Hsinchu, Taiwan).

### 2.5. Auto-Antibody ds-DNA Assay

Blood was drawn from eye orbit with a glass capillary syringe to prepare blood serum every two weeks since the first injection of lymphocytes. The auto-antibody ds-DNA was determined with titer plate technology and indirect immunofluorescence and was observed with a fluorescence microscope (BX50, Olympus, Tokyo, Japan).

### 2.6. Urinary Protein Excretion Test

Since the first injection of lymphocytes, mice of different groups were fed in metabolism cages every two weeks before the experiment. They drank and ate freely in cages. Their urine between 8:00 am and 8:00 am in the next day was then collected. The output of urine protein during 24 h was determined by means of Coomassie Brilliant Blue [11].

### 2.7. Histology Assay

Renal tissue specimens were fixed with 10% neutral Formalin. Following Paraffin embedding, 3 μm sections were then stained with HE. The total number of cell nuclei in the cross section of glomerulus was counted. The hardening exponent of the glomerulus was determined by a semi-quantitative scoring method. 25 glomeruli of each animal were counted and were averaged [11].

### 2.8. Reverse Transcription-Polymerase Chain reaction (RT-PCR) of Inflammation—Related Gene Expression in the Kidney Tissue

Total RNA from kidney tissue was isolated using Trizol reagent (Invitrogen, Carlsbad, CA, USA) according to the manufacturer’s recommendations. The RNA was digested with RNase-free DNase (Roche, Basel, Switzerland) for 15min at 37 °C and purified using a RNeasy kit (Qiagen, Hilden, Germany) according to the manufacturer’s protocol. cDNA was synthesized from 2 μg of total RNA by incubation at 37 °C for l h with avian myeloblastosis virus reverse transcriptase (GE Healthcare, Little Chalfont, UK) with random hexanucleotides according to the manufacturer’s instruction. The primers used to specifically amplify the genes of interest were: NF-κB-p65 forward: 5′-CAC TTA TGG ACA ACT ATG AGG TCT CTG G-3′ and reverse: 5′-CTG TCT TGT GGA CAA CGC AGT GGA ATT TTA GG-3′; IκB-α forward: 5′-GCT GAA GAA GGA GCG GCT ACT-3′ and reverse: 5′-TCG TAC TCC TCG TCT TTC ATG GA-3′; TGF-β1 forward: 5′-CTT CAG CTC CAC AGA GAA GAA CGT C-3′ and reverse: 5′-CAC GAT CAT GTT GGA CAA CTG CTC-3′; Fas forward: 5′-GAA ATG AAA TCC AAA GCT-3′ and reverse: 5′-TAA TTT AGA GGC AAA GTG GC-3′; FasL forward: 5′-GGA TTG GGC CTG GGG ATG TTT CA-3′ and reverse: 5′-TTG TGG CTC AGG GGC AGG TTG TTG-3′. The internal control gene of GAPDH was amplified using the primers: forward: 5′-CGG AGT CAA CGG ATT TGG TC-3′ and reverse: 5′-AGC CTT CTC CAT GGT CGT GA-3′. Amplification was performed in a thermal cycler (Eppendorf, Hamburg, Germany). The polymerase chain reaction (PCR) products were separated in 1.0% agarose gels and visualized with ethidium bromide staining [12].

### 2.9. Protein Extraction and Western Blot Analysis in the Kidney Tissue

Total kidney tissue protein was obtained with RIPA buffer as previously described. Protein concentrations were determined with a Bio-Rad protein assay kit (Hercules, CA, USA). For the western blot analysis, aliquots of the lysate containing 30–50 μg protein were separated by sodium dodecyl sulfate-polyacrylamide gel electrophoresis (SDS-PAGE) and then electrotransferred onto a nitrocellulose membrane (Schleicher and Schuell, Keene, NH, USA). The membranes were subjected to immunoblot analysis and the proteins were visualized by an enhanced chemiluminescence (ECL) method (GE Healthcare, Buckinghamshire, UK). The cell lysates were separated by 12% SDS-PAGE, transferred onto a polyvinylidene fluoride membrane (GE Healthcare, Buckinghamshire, UK), blocked with 5% skimmed milk and hybridized with primary antibodies (diluted 1:1000). The antibodies against NF-κB-p65, IκB-α, TGF-β1, Fas and FasL were obtained from Santa Cruz Biotechnology Inc. (Santa Cruz, CA, USA). The blots were then incubated with the horseradish peroxidase—conjugated secondary antibody (Santa Cruz Biotechnology Inc., Santa Cruz, CA, USA) for 1 h at room temperature. The blots were washed three times with PBS-T and then developed by enhanced chemiluminescence (Amersham Life Science, Arlington Heights, IL, USA) [12].

### 2.10. Statistical Analysis

Data are presented as the mean ± SD. Differences between the mean values for individual groups were assessed with a one-way ANOVA with Duncan’s multiple range test. Differences were considered significant when *p* < 0.05. SAS version 9.1 (SAS Institute Inc., Cary, NC, USA, 2009) was used for statistical analyses [12].

## 3. Results

### 3.1. Cytokine IL-6, IL-12, TNF-α and IFN-γ Levels

The normal mice showed the lowest IL-6, IL-12, TNF-α and IFN-γ levels; however, these levels of control mice were more than three times of normal mice (*p* < 0.05, Table 1). The levels of IL-6, IL-12, TNF-α and IFN-γ in 25 and 50 mg/kg PLYCSB treated mice were lower than control mice. And the 50 mg/kg PLYCSB treated mice had lower levels of IL-6, IL-12, TNF-α and IFN-γ than 25 mg/kg PLYCSB treated mice.

**Table 1:** Serum IL-6, IL-12, TNF-α and IFN-γ levels of lupus nephritis mice treated with polysaccharide of large yellow croaker swim bladder (PLYCSB).

Group	IL-6 (pg/mL)	IL-12 (pg/mL)	TNF-α (pg/mL)	IFN-γ (pg/mL)
Normal	51.34 ± 3.63 ^a^	432.65 ± 41.26 ^A^	110.36 ± 6.68 ^a^	41.27 ± 2.98 ^A^
Control	308.95 ± 31.23 ^b^	1711.56 ± 89.91 ^B^	731.83 ± 13.60 ^b^	127.28 ± 8.13 ^B^
PLYCSB				
25 mg/kg	201.46 ± 22.37 ^c^	1227.10 ± 77.21 ^C^	537.83 ± 19.55 ^c^	81.43 ± 5.44 ^C^
50 mg/kg	131.61 ± 20.43 ^d^	871.74 ± 32.36 ^D^	301.18 ± 14.76 ^d^	63.65 ± 6.35 ^D^

^a–d, A–D^ Mean values with different letters in the same column are significantly different (*p* < 0.05) according to Duncan’s multiple range test.

### 3.2. Serum SCr, BUN, TC, TG, TP and ALB Levels

The SCr, BUN, TC and TG serum levels in control mice were higher than in the other groups mice and these levels in normal mice were significantly decreased (*p* < 0.05, Table 2). Mice treated with PLYCSB also had decreased levels of SCr, BUN, TC and TG levels compared to the control mice, but higher than in normal mice, PLYCSB at 50 mg/kg could bring levels of SCr, BUN, TC, and TG close to normal levels. TP and ALB serum levels in this experiment showed the reverse trends, the levels in each group from high to low were the normal group, 50 mg/kg treated group, 25 mg/kg treated group and control group.

**Table 2:** Serum SCr, BUN, TC, TG, TP and ALB levels of lupus nephritis mice treated with polysaccharide of large yellow croaker swim bladder (PLYCSB).

Group	SCr (μmol/L)	BUN (mmol/L)	TC (mmol/L)	TG (mmol/L)	TP (g/L)	ALB (g/L)
Normal	61.21 ± 4.21 ^a^	1.44 ± 0.36 ^A^	1.71 ± 0.23 ^d^	0.97 ± 0.12 ^A^	52.27 ± 3.28 ^b^	35.18 ± 1.38 ^B^
Control	134.72 ± 17.88 ^b^	14.87 ± 2.12 ^B^	16.42 ± 2.17 ^a^	14.76 ± 1.28 ^B^	22.82 ± 1.65 ^a^	12.27 ± 0.22 ^A^
PLYCSB						
25 mg/kg	107.82 ± 2.86 ^c^	7.22 ± 1.18 ^C^	12.67 ± 2.97 ^b^	8.39 ± 1.26 ^C^	35.91 ± 1.88 ^d^	20.09 ± 0.98 ^D^
50 mg/kg	80.37 ± 2.77 ^d^	4.62 ± 0.87 ^D^	6.26 ± 0.67 ^c^	4.51 ± 1.19 ^D^	42.67 ± 1.20 ^c^	28.17 ± 0.68 ^C^

^a–d, A–D^ Mean values with different letters in the same column are significantly different (*p* < 0.05) according to Duncan’s multiple range test.

### 3.3. ds-DNA Positive Rate during the Experiment

The autoantibody ds-DNA was measured by indirect immunofluorescence in the 2nd, 4th, 6th, 8th, 10th and 12th week. The ds-DNA antibody titer of serum was observed with a fluorescence microscope. With the tilter being 1:20, the result shows that all mice of the control group tested positive since the sixth week and the LN induction to them all is successful. Mice from the low-concentration group tested positive at the 8th week and mice from the high-concentration group tested positive at the 10th week, which means that the samples slow down the speed that mice were infected with LN (Table 3).

**Table 3:** ds-DNA positive rate of polysaccharide of large yellow croaker swim bladder (PLYCSB) treated lupus nephritis mice (*n* = 10, titer = 1:20).

Group	2 Weeks	4 Weeks	6 Weeks	8 Weeks	10 Weeks	12 Weeks
Normal	0/10 ^a^ (0%)	0/10 ^A^ (0%)	0/10 ^d^ (0%)	0/10 ^D^ (0%)	0/10 ^c^ (0%)	0/10 ^C^ (0%)
Control	5/10 ^b^ (50%)	8/10 ^B^ (80%)	10/10 ^b^ (100%)	10/10 ^B^ (100%)	10/10 ^b^ (100%)	10/10 ^B^ (100%)
PLYCSB						
25 mg/kg	4/10 ^c^ (40%)	6/10 ^C^ (60%)	8/10 ^c^ (80%)	10/10 ^B^ (100%)	10/10 ^b^ (100%)	10/10 ^B^ (100%)
50 mg/kg	4/10 ^c^ (40%)	5/10 ^D^ (50%)	6/10 ^a^ (60%)	7/10 ^C^ (70%)	10/10 ^b^ (100%)	10/10 ^B^ (100%)

^a–d^^, A–D^ Mean values with different letters in the same column are significantly different (*p* < 0.05) according to Duncan’s multiple range test.

### 3.4. Change of Urine Protein during the Experiment

The output of urine protein in normal mice showed no significant change during the experiment, but mice from the other groups displayed increased urine protein as time prolonged. The urine protein contents in lupus nephritis mice were higher than those in PLYCSB treated mice and normal mice (Table 4). The output of urine protein in mice treated with a high concentration (50 mg/kg dose) PLYCSB was closer to the normal mice.

**Table 4:** Output of urine protein of polysaccharide of large yellow croaker swim bladder (PLYCSB) treated lupus nephritis mice (*n* = 10, mg/24 h).

Group	2 Weeks	4 Weeks	6 Weeks	8 Weeks	10 Weeks	12 Weeks
Normal	0.73 ± 0.09 ^a^	0.79 ± 0.11 ^A^	0.82 ± 0.06 ^a^	0.88 ± 0.10 ^A^	0.87 ± 0.11 ^a^	0.90 ± 0.04 ^A^
Control	5.75 ± 0.63 ^b^	6.22 ± 0.54 ^B^	7.87 ± 0.81 ^b^	8.24 ± 0.72 ^B^	8.88 ± 0.50 ^b^	9.12 ± 1.01 ^B^
PLYCSB						
25 mg/kg	3.26 ± 0.41 ^c^	4.03 ± 0.61 ^C^	4.50 ± 0.36 ^c^	5.11 ± 0.53 ^C^	6.05 ± 0.39 ^c^	6.39 ± 0.41 ^C^
50 mg/kg	2.02 ± 0.27 ^d^	2.35 ± 0.19 ^D^	2.72 ± 0.11 ^d^	3.12 ± 0.23 ^D^	3.41 ± 0.07 ^d^	4.89 ± 0.38 ^D^

^a–d, A–D^ Mean values with different letters in the same column are significantly different (*p* < 0.05) according to Duncan’s multiple range test.

### 3.5. Glomerular Number and Glomerular Sclerosis Index by Histological Analysis

In the end of this experiment, through the H&E histological analysis, there were 35 ± 3 glomeruli in one kidney section for each normal mice, the glomerular number in control mice was much lower with 13 ± 3. PLYCSB could help to reduce the glomerular change, and the 50 mg/kg dose PLYCSB showed a stronger effect (Table 5). Glomerular sclerosis index of control mice was highest in all groups, and the sclerosis indexes of PLYCSB-group mice were reduced depending on the concentration.

**Table 5:** Glomerular number and glomerular sclerosis index of polysaccharide of large yellow croaker swim bladder (PLYCSB) treated lupus nephritis mice (*n* = 10, mg/24 h).

Group	Number of Glomerular	Glomerular Sclerosis Index
Normal	35 ± 3 ^a^	0.00 ± 0.00 ^A^
Control	13 ± 3 ^b^	3.12 ± 0.96 ^B^
PLYCSB		
25 mg/kg	22 ± 2 ^c^	2.03 ± 0.45 ^C^
50 mg/kg	27 ± 3 ^d^	1.57 ± 0.32 ^D^

^a–d^^, A–D^ Mean values with different letters in the same column are significantly different (*p* < 0.05) according to Duncan’s multiple range test.

### 3.6. Gene Expression of NF-κB-p65, IκB-α, TGF-β1, Fas and FasL

**Figure 1:**
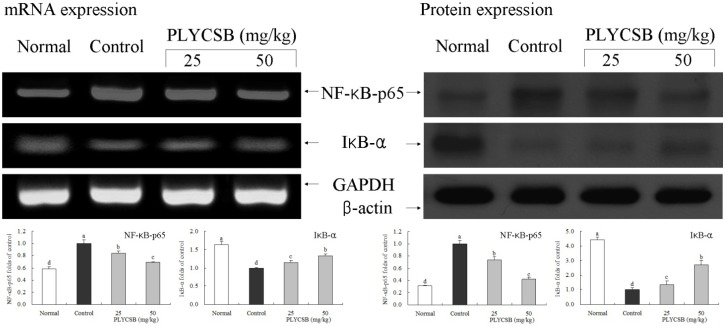
Effects of large yellow croaker swim bladder (PLYCSB) on the mRNA and protein expression of NF-κB-p65 and IκB-α in lupus nephritis mice. Fold-ratio: gene expression/GAPDH (β-actin) × control numerical value (control fold ratio: 1). ^a–d^ Mean values with different letters over the bars are significantly different (*p* < 0.05) according to Duncan’s multiple range test.

**Figure 2:**
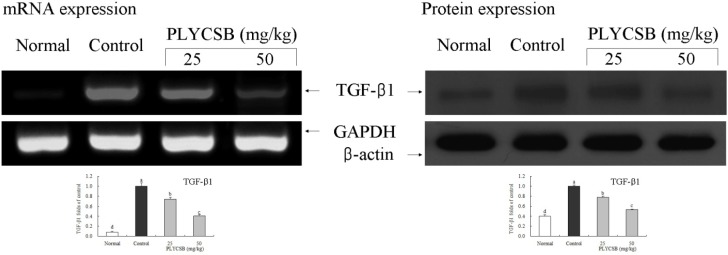
Effects of large yellow croaker swim bladder (PLYCSB) on the mRNA and protein expression of TGF-β1 in lupus nephritis mice. Fold-ratio: gene expression/GAPDH (β-actin) × control numerical value (control fold ratio: 1). ^a–d^ Mean values with different letters over the bars are significantly different (*p* < 0.05) according to Duncan’s multiple range test.

**Figure 3:**
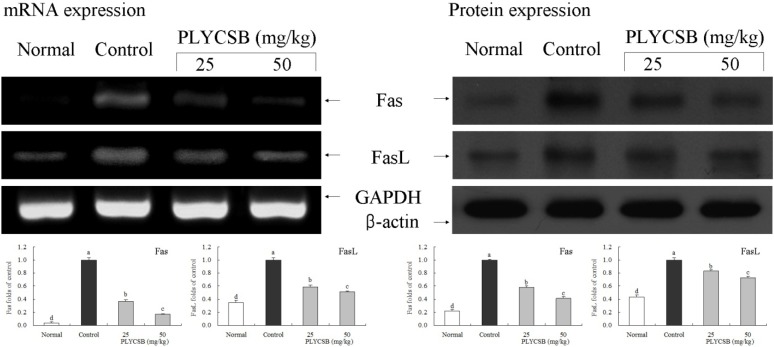
Effects of large yellow croaker swim bladder (PLYCSB) on the mRNA and protein expression of Fas and FasL in lupus nephritis mice. Fold-ratio: gene expression/GAPDH (β-actin) × control numerical value (control fold ratio: 1). ^a–d^ Mean values with different letters over the bars are significantly different (*p* < 0.05) according to Duncan’s multiple range test.

RT-PCR and western blotting were used to determine whether the inflammatory actions of PLYCSB were associated with inhibition of NF-κB-p65, IκB-α, TGF‑β1, Fas and/or FasL gene expression. As shown in Figure 1, mRNA and protein expression of NF-κB-p65 and IκB-α were changed in mice treated with PLYCSB. mRNA and protein expression of NF-κB-p65 were significantly decreased while IκB-α mRNA and protein levels were increased (*p* < 0.05). PLYCSB significantly decreased mRNA and protein expression of TGF-β1, and the TGF-β1 level mice treated with the high concentration of 50 mg/kg PLYCSB lower than those treated with the 25 mg/kg dose (Figure 2). With the PLYCSB treatment, mRNA and protein expressions of Fas and FasL were gradually elevated (Figure 3). NF-κB-p65, TGF-β1, Fas and FasL gene expression of normal mice showed the lowest levels, control mice showed the highest levels, and the IκB-α of normal and control mice showed the opposite tendency. Overall, the results of this experiment showed that PLYCSB had a potent anti-inflammatory effect on lupus nephritis.

## 4. Discussion

Swim bladder has been historically used as a Chinese folk medicine. Base on previous scientific data, swim bladder has been recently reported to ameliorate different pathological conditions of inflammation, as well as effectively strengthening the function of platelets, capillary vessels and clotting factors [13]. Polysaccharide is the main constituent of swim bladder, but there were not many studies performed regarding the polysaccharide found in the swim bladder. In this study, the therapeutic effect on lupus nephritis of polysaccharide of large yellow croaker swim bladder was determined in mice, and the results demonstrated that the PLYCSB conferred a therapeutic effect of lupus nephritis in animal model experiments.

Serum creatinine and urea nitrogen are nitrogenous organic compounds and the end products of protein metabolism. When renal function was normal, these small molecules are filtered from the glomerulus. When the kidney suffers from lesions, the filtering capability of the glomerulus decreased and the content of serum creatinine and urea nitrogen increased. This increase in serum levels could be used as an index for the clinical diagnosis of renal damage [14].

Too much cholesterol and triglycerides are the cause hyperlipemia. When nephropathy deteriorated to a certain degree, features of hyperkalemia would co-exist. Therefore, cholesterol and triglyceride could also be considered as an index to renal hypofunction and renal lesion [15].

Protein in urine was lost for a long time in nephrotic syndrome. The total protein in serum was drastically reduced [16]. Albumin is the most prevalent protein in serum. It serves to cure serious diseases including edema caused by clinically curative nephropathy. It was thus evident that keeping the amount of total protein and albumin in serum is an important way to maintain normal renal function. The decrease of the content of total protein and albumin are the manifestations of poor renal function [15].

The final stage of lupus nephritis is represented by Global sclerosis involving most of the glomeruli, and represents healing prior to inflammatory injury. The sclerosis aggravated glomeruli parenchymal cells reducing [17].

A high-concentration of antibody to ds-DNA seemed to be seen only in SLE. Therefore, this antibody to ds-DNA was specific to SLE. The antibody to ds-DNA was closely related to the disease activity index and especially to LN and could be used as an index to diagnose systematic lupus erythematosus [18].

NF-κB is an important transcription factor serving to convey signals in the cell. It’s reported that it is decisive in the gene transcription regulation of numerous factors in the aforementioned immune inflammation [19]. Nephridial tissue of normal mice appeared with a few mesangial cell and NF-κB, which indicates that moderate NF-κB activity might be necessary to adjust physiological function of normal mixed renal cells [20]. Guijarro *et al*. [21] found that activation of NF-κB in mesangial cell nurtured *in vitro* can enhance the reproduction of mesangial cell and serves to fulfill centering control function to multiple chemokines secreted by mesangial cells. According to Rangan *et al*. [22], when interstitial nephritis was accompanied by large amount of albuminuria, NF-κB in the renal cortex was activated and TNF-α, IL-6 and TGF-β increased. After treatment, the activation of NF-κB decreased and damage of renal interstitium decreased, and IL-6 and TGF-β expression reduced. By the expression quantity of TGF-β mRNA in kidneys of the BXSB mice assay, TGF-β played an important role in the pathological accumulation of extracellular matrix in lupus nephritis and the formation process of glomerular sclerosis [23]. Experimental results showed that TNF-α and IL-6 levels in serum of the PLYCSB treated groups decrease greatly compared with the LN control group, which came to the same result with that of Rangan. IL-12 plays an important role in LN autoimmune response, and the level of IL-12 in the active stage of LN rises. One of the features of LN was the emergence of large amounts of autoantibody. It was proven that IL-12 can promote cells to produce this autoantibody directly. The increase in the content of IL-12 led to the large amount of production of this autoantibody [24]. As an inflammatory mediator, IFN-γ takes part in the whole immune inflammatory process of nephritis. The level IFN-γ in glomerulonephritis is substantially increased [25]. After the occurrence of nephritis, cytokines related to inflammation change. The content of cytokines related to inflammation, like IL-6, IL-12, TNF-α and IFN-γ, in blood would also obviously increase [26]. PLYCSB serves to reduce the content of these cytokines in blood. Thereby, PLYCSB has an obvious curative effect to LN. In this research, the expression of NF-κB and TGF-β in mice in the LN control group is higher than that in mice of the normal group and the PLYCSB treated groups. The better the curative effect of PLYCSB, the weaker the expression of NF-κB and TGF-β. Meanwhile, IκB expression showed the opposite tendency.

Fas and FasL are the molecules that cause the death of cells. Fas is a member of the ErbB of TNF nerve growth factors and exists in the surface of active T/B leukomonocytes and hematopoietic cells, as well as other tissues [27]. Fas induced the death of cells through oxidative stress after interacting with its natural ligand [28]. It was proven that the apoptosis rate of monocytes of LN patients is faster than that of normal people. FasL is a member of TNF family. In clinical checks, it was found that no FasL is obviously detected in the tissues of normal people while there is palpable FasL in LN patients. The increase of FasL level is likely to be caused by the change of LN [29].

## 5. Conclusions

In summary, the lupus nephritis therapeutic effect of PLYCSB was evaluated by various *in vivo* experimental methods, including serum cytokine assays of IL-6, IL-12, TNF-α and IFN-γ; an assay for serum levels of SCr, BUN, TC, TG, TP and ALB; an auto-antibody ds-DNA assay, analysis of urinary protein excretion, a histology assay, tissue RT-PCR and western blot assays for checking the inflammatory related genes of NF-κB-p65, IκB-α, TGF‑β1, Fas and FasL. Analysis of the various mice treatment groups revealed that PLYCSB had a therapeutic effect on lupus nephritis, indicating that PLYCSB represents a potentially useful agent for the treatment or prevention of lupus nephritis in mice.

## References

[1] Jian J.C., Wu Z.H. (2003). Effects of traditional Chinese medicine on nonspecific immunity and disease resistance of large yellow croaker, *Pseudosciaena crocea* (Richardson). Aquaculture.

[2] Li C., Yao C.L. (2013). Molecular and expression characterizations of interleukin-8 gene in large yellow croaker (*Larimichthys crocea*). Fish Shellfish Immunol..

[3] Liu S., Yu B. (2009). Peptides from variegated carp (*Aristichthys nobilis*) swim bladder: Fermentation production and assessment of antioxidant properties. Food Sci..

[4] Yu Z.H., Yin L.H., Yang Q., Liu Y. (2009). Effect of *Lentinus edodes* polysaccharide on oxidative stress, immunity activity and oral ulceration of rats stimulated by phenol. Carbohydr. Polym..

[5] Laurienzo P. (2010). Marine polysaccharides in pharmaceutical applications: An overview. Mar. Drugs.

[6] Zhang J.J. (2008). Progress in diagnosis and treatment of lupus nephritis. Acad. J. Kunming Med. Coll..

[7] Ma H.L., Zhang X.G., Zhang X.Z., Xu Y. (2012). Effect of esculentoside A on therapeutic and cytokines secretion of lupus nephritis in BXSB mice. Chongqing Med. J..

[8] Bruijn J.A., van Elven E.H., Hogendoorn P.C., Corver W.E., Hoedemaeker P.J., Fleuren G.J. (1988). Murine chronic graft-*versus*-host disease as a model for lupus nephritis. Am. J. Pathol..

[9] Yan C., Yan X. (2008). Study on extraction of *Lycium barbarum* polysaccharides by different methods and their antioxidant effects *in vitro*. Food Sci..

[10] Bo Y.H., Jiang F., Su W., Ling Y.L., Bi Z.Q. (2007). The protective effect of idiotypic peptide on kidney in the GVHD lupus nephritis mice. J. Cap. Med. Univ..

[11] Zhao X., Kim S.Y., Park K.Y. (2013). Bamboo salt has *in vitro* anticancer activity in HCT-116 cells and exerts anti-metastatic effects *in vivo*. J. Med. Food.

[12] Chen L.H., Song J.L., Qian Y., Zhao X., Suo H.Y., Li J. (2014). Increased preventive effect on colon carcinogenesis by use of resistant starch (RS3) as the carrier for polysaccharide of *Larimichthys crocea* swimming bladder. Int. J. Mol. Sci..

[13] Cao H., Tian X.L., Liu X. (2009). Study on molecular identification and pharmacology of hemostasis action for isinglass. J. Chin. Inst. Food Sci. Technol..

[14] Li X.Y., Kong F.Y., Zhang H.Q., Tang R.X., Zheng K.Y. (2011). The duplication and identification of anti-glomerular basement membrane (GBM) nephritis model in mice. Acta Acad. Med. Xuzhou.

[15] Chou A., Zhou J.Y., Zhou Y., Hua J., Wu J.B. (2012). Therapeutic effect of zhenwu decoction on chronic glomerulonephritis rat model induced by cationization bovine serum albumin osmotic pump. Tradit. Chin. Drug Res. Chin. Pharm..

[16] Chen W. (2010). Changes of urine protein in patients with chronic glomerulonephritis after different doses of irbesartan treatment. Chin. J. Arterioscler..

[17] Du J., Wang Q.S., Jia R.H. (2005). Glomerular cell proliferation and apoptosis experimental glomerulosclerosis. J. Pract. Med..

[18] Wang M.F. (2010). Significance of anti-double stranded DNA in system lupus erythematosus. Chin. J. Health Lab. Technol..

[19] Philip S., Bulbule A., Kundu G.C. (2004). Matrix metalloproteinase-2: Mechanism and regulation of NF-κB-mediated activation and its role in cell motility and ECM-invasion. Glycocorj. J..

[20] Yao C.W., Tang D.S., Liang D. (2005). Expression and significance of NF-κB in mouse renal tissue with lupus nephritis-prone BXSB. J. Guangdong Med. Coll..

[21] Guijarro C., Kim Y., Kasiske B.L. (1997). Central role of the transcription factor NF-κB in mesangial cell production of chemokines. Contrib. Nephrol..

[22] Rangan G.K., Wang Y., Tay Y.C., Harris D.C. (2000). Cytokine gene expression in Adriamycin nephropathy: Effects of antioxidant nuclear factor κB inhibitors in established disease. Nephron.

[23] Zhou P., Chen X.R., Li S.M., Tu S.Q. (1999). Expression of transforming growth Factor-β genes in kidneys of BXSB lupus mice. Chin. J. Dermatol. Venereol..

[24] Li Z.J., Li Y.J., Yang Q.Q., Yang X., Xu Y.W., Yu X.Q. (2002). Significance of levels IL-12 and IgG in patients with lupus nephritis. J. New Med..

[25] Xiang L., Gao X.X., Pan J.R. (2006). Serum levels of interferon-gamma and interleukin-10 in patients with chronic glomerulonephritis and their clinical significance. Clin. Med. J. China.

[26] Wang Q., Sun P., Li G.J., Zhu K., Wang C., Zhao X. (2014). Inhibitory effects of Dendrobium candidum Wall ex Lindl. on azoxymethane- and dextran sulfate sodium-induced colon carcinogenesis in C57BL/6 mice. Oncol. Lett..

[27] Takahashi T., Tanaka M., Inazawa J., Abe T., Suda T., Nagata S. (1994). Human Fas ligand: Gene structure, chromosomal location and species specificity. Int. Immunol..

[28] Itoh N., Yonehara S., Ishii A., Yonehara M., Mizushima S., Sameshima M., Hase A., Seto Y., Nagata S. (1991). The polypeptide encoded by the cDNA for human cell surface antigen Fas can mediate apoptosis. Cell.

[29] Jiang J.P., Yi Z.H., Liu Z.R., Hu L.P., Hou F.F. (2001). Expression Fas on monocytes and plasma soluble FasL in patients with lupus nephritis. Chin. J. Rheumatol..

